# A systematic process to formulate quality lessons learned about hospital resilience during the COVID-19 pandemic

**DOI:** 10.1080/16549716.2025.2547439

**Published:** 2025-09-19

**Authors:** Roula Haddad, Christian Dagenais, Muriel Kielende, Aurélie Hot, Valéry Ridde

**Affiliations:** a Department of Psychology, Université de Montréal, Montreal, Quebec, Canada; b School of Public Health, Université de Montréal, Montreal, Quebec, Canada; c Université Paris Cité, IRD, Inserm, Ceped, Paris, France

**Keywords:** Quality lessons learned, public health, Healthcare, HoSPiCOVID, knowledge transfer

## Abstract

**Background:**

The urgency of the COVID-19 pandemic forced hospitals to adjust swiftly to the health crisis. They adopted a variety of solutions and encountered numerous challenges. To enhance hospitals’ readiness for future crises, the HoSPiCOVID research project documented the experiences of seven hospitals in five countries (Brazil, Canada, France, Japan, Mali) to extract quality lessons learned (QLLs). These were subsequently refined through workshops involving healthcare professionals.

**Objectives:**

The aim of this study was to examine the process of conducting QLL formulation workshops in the HoSPiCOVID hospitals to identify facilitators and barriers encountered.

**Method:**

This study was based on a qualitative approach. Semi-structured individual interviews (*N* = 13) were conducted with researchers and professionals in the five countries. Interview recordings were imported into NVivo software, transcribed, and thematically analyzed.

**Results:**

Although the professionals participated actively in the workshops, group dynamics were sometimes impeded by existing power dynamics among participants. Nonetheless, professionals perceived the workshops as an optimal method for formulating QLLs. Distributing summary sheets of workshop content beforehand and ensuring the alignment of content with participants’ needs enhanced the effectiveness of the process.

**Conclusion:**

Despite obstacles encountered in the workshops, participants appreciated the initiative of documenting QLLs using a structured approach in which the QLLs were refined and professionals were able to compare experiences. The QLLs formulated could improve hospitals’ responses to future health crises. The recommendations from this study could also enhance the organization of future workshops aiming to formulate QLLs.

## Paper Context

- **Main findings:** Despite the presence of factors impeding the workshops’ conduct, this method was found to be optimal for formulating Quality Lessons Learned.
- **Added knowledge:** Taking into account the facilitators and barriers identified, this paper will enable the improvement of future Quality Lessons Learned documentation initiatives.
- **Global health impact for policy and action:** The results will enable the formulation of precise guidelines to follow when conducting Quality Lessons Learned documentation initiatives, particularly during times of health crises.

## Background

An infectious disease caused by the severe acute respiratory syndrome coronavirus 2 (SARS-CoV-2) was detected in China in December 2019 [1]. The virus quickly spread to various countries, prompting the World Health Organization (WHO) to declare it a public health emergency on 30 January 2020, and classify it as a pandemic on 11 March 2020 [1]. Hospitals had to adapt to this health crisis and handle the surge in hospitalization rates [2].

In this context, the HoSPiCOVID research project sought to explore and compare the resilience of seven hospitals across five countries (Brazil, Canada, France, Japan, Mali) in response to the pandemic [3]. The primary objective of this project was to identify the challenges encountered by healthcare workers in these hospitals, with the overarching goal of drawing quality lessons learned (QLLs) to enhance preparedness for future health crises. In accordance with WHO guidelines, the process involved gathering feedback (*After Action Review*) from healthcare professionals to formulate QLLs [4].

QLLs are defined as statements or recommendations based on information obtained through tacit or explicit experience and knowledge, aimed at enhancing a process or an activity [5]. Our team conducted a rapid review to determine the steps to be followed when formulating QLLs [6]. That review identified 11 steps divided into two phases [6]:

– Phase 1, preparatory steps: 1) identify and engage stakeholders; 2) formulate the objective(s) of the process; 3) identify the projects to be considered; 4) select the time for developing QLLs; and 5) choose the data collection strategy.
– Phase 2, steps to identify and formulate QLLs: 6) select data collection tools; 7) collect the data; 8) verify the data; 9) analyze and formulate QLLs; 10) check QLL quality; and 11) archive, disseminate, and implement QLLs.

After the completion of Phase 1 [5], individual interviews were carried out with healthcare professionals from the project’s participating hospitals (Table 1). These interviews served to gather preliminary data on hospitals’ resilience and functioning during the pandemic, leading to the formulation of preliminary QLLs. These QLLs covered four themes common to all five countries: 1) infrastructure reorganization; 2) human resources management; 3) infectious risk prevention and control; and 4) logistics and supply [5]. The professionals were then introduced to the QLLs through summary sheets and were invited to participate in a workshop. Using a visually appealing format tailored to the target audiences, the summary sheets grouped together the preliminary formulated QLLs of each country. The aim of the workshops was not only to discuss the QLLs initially formulated, but also to complete, improve, and validate them. In Mali, Brazil, and France, the workshops were conducted in person, whereas in Canada they were conducted online. The workshops were held with representatives from all hospitals except those of Japan, due to the increase in admissions of patients with COVID-19. In Japan, rather than attending workshops, professionals were interviewed individually. The sequence of activities leading to the formulation of QLLs is shown in Table 1.

**Table 1.** The sequence of activities for QLL formulation.

Activity 1	Individual interviews with hospital professionals and development of preliminary QLLs
Activity 2	Distribution of summary sheets containing the preliminary formulated QLLs
Activity 3	Workshops with professionals to complete, improve, and validate the QLLs initially formulated; in Japan, individual interviews conducted as a substitute for workshops

Our aim in this study was to analyze and examine the QLL formulation workshops and to identify the facilitators and obstacles encountered.

## Methods

This study followed a qualitative approach that was both exploratory and comparative. The exploratory method was used to describe how the QLL formulation workshops were conducted. For the comparative method, data collected from professionals and researchers were analyzed and compared. The qualitative approach will be presented here based on the COREQ (COnsolidated criteria for REporting Qualitative research) checklist [7].

### Procedure

To evaluate the QLL formulation workshops, semi-structured individual interviews lasting 30–45 minutes were conducted with researchers and professionals. The interviews were conducted on Zoom by one of the coauthors (MK) and in French: transcripts were loosely translated into English.

### Participants selection process

A convenience sampling method was used. This non-probabilistic method involves selecting people based on their accessibility and involvement in the process [8]. In some countries, two researchers agreed to take part in the interviews (Mali, Canada, and France), while in others, only one researcher was recruited (Japan and Brazil). Each researcher proposed two key professionals to take part in the interviews.

The names of the hospitals in which the researchers (R) (*N* = 8) and professionals (P) (*N* = 5) were recruited were anonymized: Mali (two researchers; one professional), Hospital 1 in Canada (one researcher; one professional), Hospital 2 in Canada (one researcher), France (two researchers; three professionals), Brazil (one researcher), and Japan (one researcher). Professionals recruited were department heads, senior managers, hospital practitioners (i.e. physicians and nurses), and prevention officers.

### Data collection tools

The interviews were based on two separate interview grids. Some questions were derived from the QLL formulation guide [6]. The interviews with researchers covered the following themes:

– Quality of the information gathered and characteristics of the QLL formulation workshops (i.e. time and place, professionals’ attitudes, group composition and dynamics, workshop modality and structure, themes addressed).
– Factors that helped and hindered the process (e.g. strategy and timing of data collection, themes covered, workshop participants).
– Recommendations from researchers to improve the documentation of future QLLs in similar contexts.

Interviews with professionals focused on the following points:

– Perceptions of the QLL formulation initiative and its characteristics (relevance, timing, form, group composition, themes addressed, workshop facilitation).
– Professionals’ views regarding the potential of QLLs to provide operational guidance for more effective crisis response.
– Optimal forms of QLL dissemination and main target audiences.
– Factors facilitating and hindering the dissemination and use of formulated QLLs.
–Recommendations from professionals to improve the QLL formulation process (e.g. in terms of strategy or timing of data collection, themes addressed, and group composition).

### Data analysis

The audio recordings of the interviews were first imported into NVivo software and organized into two categories: researchers and professionals. They were then partially transcribed; partial transcription involves breaking down the interview into segments and noting the main ideas of each segment [9]. These notes can be reread to access the recording and listen to the respondent’s statements again. All interviews were subjected to thematic qualitative analysis based on an inductive approach: interview segments were open-coded without relying on pre-established codes, allowing the main themes to emerge [10]. Subsequently, the results of the interviews were interpreted to attribute meaning to the themes and to fulfill the exploratory purpose of the study. Finally, the results for the two groups of participants were compared to identify convergences and divergences. The analysis of the interviews was not intended to validate or refute a theory, but rather to provide an in-depth study of the phenomenon in question.

## Results

Four themes emerged from the interviews’ qualitative analysis (*N* = 13): 1) factors influencing group dynamics during the workshops; 2) factors contributing positively to the workshops; 3) factors contributing to the utility of the workshops and of the information gathered; 4) and recommendations for improving future QLL formulation workshops. The case of Japan was not included in the analysis of the first theme because individual interviews were conducted with professionals rather than workshops.

### Theme 1: factors influencing group dynamics during the workshops

Participants highlighted various factors that either facilitated or impeded group dynamics and the professionals’ engagement during workshops. Building a trusting relationship between researchers and professionals (*n* = 2) and the active involvement and engagement of professionals during the workshops (*n* = 7) positively influenced the group dynamics. The heterogeneous samples of participants (i.e. with different hierarchical statuses) not only ensured the broad representation of professionals but also encouraged them to share perspectives and collaborate on addressing potential challenges (*n* = 3).

However, the diversity within the groups also presented challenges. In some instances, the presence of professionals with varying hierarchical positions led to power dynamics (*n* = 5) and frequent interruptions of professionals’ statements (*n* = 3). This sometimes resulted in professionals’ being less willing to participate in discussions and share their own experiences during the workshop (*n* = 6): ‘*[the members] of the hospital staff who attended were trying to monopolize all the conversation for themselves*’ (R-Mali-2); *‘If there are a lot of people with a lot of differences [in levels] of authority, I think that’s one thing that can bias the workshop a lot…. At one point, the director [present] showed her authority over the others [i.e. her colleagues]…. A person like her can make others not want to talk*’ (R-Brazil).

Despite the presence of certain obstacles hindering the workshops, the professionals felt this activity was optimal for formulating QLLs (*n* = 3). The workshops encouraged direct exchanges between professionals, the free sharing of their experiences, and the exploration of their personal and individual experiences during the pandemic: *‘In this traumatic experience, everyone has their own story, and we each need to tell our own story…. In this respect, the workshops were essential, allowing each person to express themselves*’ (P-Fr-2). ‘*People speak much more freely in these groups than in the more formal feedback sessions*’ (P-Fr-1).

### Theme 2: factors contributing positively to the workshops

Participants identified two factors that had a positive impact on the workshops: the professionals’ appreciation of the researchers’ rigorous attitude (*n* = 4) and the effective organization of the workshop activities (*n* = 3). The researchers were thoroughly prepared to guide the workshops and had a strong grasp of the subject matter and topics to be discussed, which helped establish their credibility with the professionals. Nevertheless, certain challenges hampered workshop facilitation, including issues related to time management and the need for some researchers to become more familiar with the process (*n* = 3).

The relevance of the content also had a beneficial impact on the workshop, as it was aligned with the professionals’ concerns and real-life situations. The distribution of summary sheets on the themes being discussed helped professionals be informed and better prepared, which potentially encouraged their participation (*n* = 2). However, a researcher in Mali pointed out that the content should have been formulated more clearly for professionals: *‘I think we need to lighten the content, that is, make the terminology much more understandable to hospital staff. Perhaps we were using terms that weren’t entirely clear to them*’ (R-Mali-1). The professionals’ involvement in content creation appeared to have increased their interest in the transferred content (*n* = 1). Professionals appreciated being able to derive practical recommendations from the workshop’s content (e.g. equipment storage, continuing education, patient discharges). They stressed the importance of evaluating whether these recommendations were subsequently implemented by the hospitals or if the habitual practices were reinstated (*n* = 3).

Regarding the timing of the workshops, a temporal issue arose (*n* = 5). Because information was continuously evolving over the various pandemic waves and the workshops were not held immediately after the initial interviews with professionals, the topics discussed in the workshops sometimes no longer aligned with the current reality in the field; they needed to be updated. Some researchers suggested that the workshops’ timing should have been reconsidered to accommodate the professionals’ limited availability and heavy workloads (*n* = 5): ‘*We had trouble recruiting enough people to take part [in the workshops]. I think it wasn’t a very easy time, either, as the hospital was facing a [new] wave of the pandemic*’ (R-CA-1).

### Theme 3: factors contributing to the utility of the workshops and of the information gathered

No challenges were identified with respect to the quality of the information gathered in the workshops. The workshops enabled researchers to delve more deeply into the data gathered from professionals in the initial interviews (Table 1; step 1) and to complete the formulation of the QLLs, a task that would have been more difficult to accomplish in other, more formal contexts. The participants *‘…brought back information that was extremely relevant to us from a research standpoint. This information enabled us to fill in the gaps*’ (R-Mali-1). The workshops facilitated in-depth discussions on diverse themes and took into account professionals’ varied experiences during the pandemic. These discussions highlighted concerns at both the individual (e.g. perception that healthcare providers were not sufficiently protected) and professional (e.g. stocks of materials and equipment) levels.

By implementing a comparative approach across countries (*n* = 2), the data collection strategy was optimized. Participants were able to explore their differences and similarities: *‘…what helped was having points to discuss with colleagues from other countries. Even if we don’t necessarily have the same lessons learned, it helped us to better reflect on the process*’ (R-CA-2). In France, when professionals spoke, researchers took the time to transcribe their statements onto a screen; this encouraged active engagement by demonstrating that their views were taken seriously (R-Fr-1). In Mali, data collection was supplemented by further interviews with professionals post-workshop, which enriched the data on QLLs (R-Mali-1).

Finally, professionals suggested that the QLLs should be distributed to four key recipient groups: 1) local authorities, to inform the decision-making process; 2) hospital management, who are responsible for the measures to be implemented, to inform them of professionals’ experiences during the pandemic; 3) all hospital departments, to compare experiences and enrich the QLLs formulated; and 4) the general public. The forms of dissemination favored by the professionals were: 1) summary fact sheets in electronic format distributed via reputable networks; 2) the media; and 3) interactive forums (e.g. meetings, seminars, symposiums).

### Theme 4: recommendations for improving future QLL formulation workshops

The researchers and professionals put forward recommendations that could help improve future QLL formulation workshops.

#### Recommendation 1: improve group composition and ensure equal participation of professionals

While it is crucial to recruit professionals from diverse hierarchical positions within hospitals, it is equally important to address power dynamics and prevent monopolization of discussions by senior staff (*n* = 4). To promote a more balanced expression of views, researchers proposed organizing workshops based on professional status and/or providing clear guidelines on workshop conduct (*n* = 3). Other strategies to foster discussion during the workshops included using credible facilitators and emphasizing the value of active participation (*n* = 2).

#### Recommendation 2: co-create and re-contextualize workshop content

Researchers suggested simplifying the formulation of the workshops’ content and focusing more directly on the interests of professionals (*n* = 5). Care should be taken to ensure participants are able to react to the selected themes and that the content is focused on gradual rather than radical changes. To facilitate their adoption, the QLLs shared with professionals should not require major changes in their workplace. Involving professionals in content creation will ensure the themes are aligned with on-the-ground realities and their goals, needs, and expectations (*n* = 5).

#### Recommendation 3: disseminate QLLs before and after workshops

Disseminating the workshop themes in advance through summary sheets was deemed an effective strategy. However, they should be distributed sufficiently in advance to give professionals time to consult them. This ensures participants are well-informed about the workshop content and can come prepared, thereby fostering interest and commitment. The QLL summary sheets should also be shared with local authorities (e.g. Ministry of Health), hospitals, and the general public (*n* = 2).

## Discussion

The aim of this study was to describe how the QLL formulation workshops were conducted and to evaluate the relevance of this data collection method. Adherence to the steps identified by Dagenais et al. [6] for QLL formulation contributed effectively to the workshop process. In addition, the basic guiding principles for QLL development were applied: creating a climate of trust, selecting appropriate facilitators, and employing a scientific approach [6]. Overall, the implementation of workshops, and thus of a strategy based on interaction, seems to have fostered productive exchanges between researchers and professionals, as well as the establishment of a relationship of trust between them.

Despite the relevance of the selected themes, the rapid changes in information during the pandemic necessitated ongoing adaptation of workshop content to real-time circumstances. The studies showed that, even if participants appreciated the quality of a workshop’s content, they still recognized the need for more updates [11]. Involving professionals in the knowledge production process not only encouraged content updating but also positioned them as knowledge co-producers and fostered better knowledge uptake and use [11–13]. Professionals emphasized the usefulness of the QLL documentation initiative and the sharing of operational guidance at the practice level. In fact, participants perceived workshop content as more useful when it offered actionable steps and was aligned with their needs and interests [11,14]. This made it possible to present clear, well-defined approaches and to promote the use of the data being transferred [12,15].

While professionals participated actively during workshops and the group dynamics generally remained positive, the heterogeneity within groups sometimes resulted in the emergence of power dynamics. These power dynamics, influenced by the diverse hierarchical statuses, had the potential to hinder the workshop process [14,16,17]. Drawing from their experiences in organizing policy dialogue workshops in West Africa, Ridde and Dagenais [18] stressed the importance of managing power dynamics that may arise during such events. They noted that individuals in higher positions tend to dominate speaking time and that their statements are often accepted without challenge, even if they are inaccurate or lacking in evidence [18]. To circumvent these power dynamics, it is advisable to pay attention to the hierarchical positions of stakeholders attending the workshop [17,19,20].

Although some professionals initially hesitated to share their experiences during the QLL documentation workshops, ultimately this strategy enabled them to compare their views through direct, informal exchanges. The researchers’ rigorous approach and the well-organized facilitation significantly contributed to the workshops’ success and the development of a trusting relationship between professionals and researchers. This relationship of trust, which is essential for promoting the use of knowledge, can be hampered by a lack of interaction [12,15,21]. Thus, the value of workshops also lies in their ability to increase interaction between researchers and professionals, and consequently to foster a climate of trust and ultimately improve knowledge use [11,15]. Several reviews of the scientific literature have highlighted the impact of trust within a team on the motivation and performance of professionals [22,23].

Disseminating summary sheets of workshop themes in advance was considered a helpful strategy. This initiative is comparable to the distribution of policy briefs before a deliberative dialogue, a practice supported by research [13,18,24]. This strategy is notable for its ability to deepen the audience’s understanding of the subject matter. It enables participants to become more familiar with the knowledge, which will eventually lead to greater knowledge assimilation and use [24].

In the present study, the researchers recruited for the evaluation interviews were representative of all those who participated in the workshops, which helped to achieve data saturation. However, there was a lower participation rate among professionals in the interviews. Furthermore, unlike the interviews conducted with researchers from the five countries in the HoSPiCOVID project, no interviews were conducted with professionals from Brazil or Japan. In Japan, ethical issues prevented the researcher from identifying key professionals, while in Brazil, language barriers hindered the recruitment of professionals, and consequently, the professionals recruited were not representative of all those who had participated in the workshops. Finally, a social desirability bias may also have influenced the interviews with both target audiences.

Based on this study, the following recommendations are proposed for researchers planning future QLL documentation workshops:

– Foster a trusting relationship between researchers and professionals.
– Provide participants with opportunities to share their experiences and perspectives, and to compare them with others.
– Address power dynamics to overcome any reticence participants may have about sharing their experiences.
– Give careful attention to the workshop’s structure and timing.
– Deliver content that resonates with participants’ concerns and offers practical guidance.
– Implement a comparative approach across countries, if feasible.
– Balance group composition and ensure equitable participation of professionals.
– Develop workshop content collaboratively with participants.
– Disseminate QLLs before and after the workshop using summary sheets.

## Conclusion and future research avenues

Both researchers and professionals appreciated the initiative to formulate QLLs through workshops. Following a scientific and rigorous approach appeared to significantly enhance the process [6]. Despite challenges encountered during the workshops, such as power dynamics and timing constraints, the QLL formulation initiative was perceived as relevant due to the valuable and informative data collected. These QLLs could potentially improve preparedness for future pandemics. During a health crisis, the content transferred in a QLL documentation initiative needs to be aligned with the rapidly changing circumstances. Several recommendations could be adopted to enhance workshop utility, such as ensuring appropriate group composition, managing power dynamics among participants, and disseminating summary sheets about the QLLs. In future research, it would be advisable to delve deeper into the factors that influence QLL documentation initiatives and to establish more precise guidelines to follow.
